# Supplementary material for: The physical and psychological well-being after a pulmonary embolism across age and comorbidities – Evidence from focus group interviews
Source: PLoS One. 2026 Apr 16;21(4):e0345551. doi: 10.1371/journal.pone.0345551 (PMC13086299; doi:10.1371/journal.pone.0345551)
Supplement: S1 Table — (DOCX) [file pone.0345551.s001.docx]

# Supplementary

## Table 1

| **Theme** | **Question** |
| --- | --- |
| Psychosocial well-being | - How did you experience being discharged and getting back to everyday life?   1. Has the PE changed your everyday life?   2. Has the PE given you any worries?   3. What have you done to get back to everyday life? |
| Physical well-being | - How has it been being physically active after the PE?   1. Does it feel different to be physically active after the PE? |
| Anticoagulation therapy | To those patients that take anticoagulation therapy?   - - How has it been taking anticoagulation therapy?   To those patients that **do not** take anticoagulation therapy?   - - How has it been having to stop anticoagulation therapy? |
| Need for rehabilitation | - - • What kind of support did you think was really good, here I am thinking of things like conversations with medical doctors, nurses, offers for exercise etc.?   - • What kind of support did you lack? |
