# Supplementary material for: The physical and psychological well-being after a pulmonary embolism across age and comorbidities – Evidence from focus group interviews
Source: PLoS One. 2026 Apr 16;21(4):e0345551. doi: 10.1371/journal.pone.0345551 (PMC13086299; doi:10.1371/journal.pone.0345551)
Supplement: Attachment — Submitted filename: Noha review.docx [file pone.0345551.s003.docx]

This study provides valuable qualitative insights into the lived experiences of PE patients, particularly the interplay between age, comorbidities, and post-PE well-being. The findings have significant potential to inform patient-centered care strategies and highlight critical gaps in post-PE rehabilitation.

**Major Concerns and Recommendations**

1. Sample Size and Composition

- Comment: Small sample size (n=17), particularly for subgroups (e.g., 2 cancer patients, 1 pregnancy-related PE), limits transferability. The single participant focus group deviates from the standard focus group methodology, potentially reducing data richness.

- Recommendation: Acknowledge these limitations more explicitly in the discussion. Despite being singular, consider adding a statement on how the pregnancy-related PE case aligns with broader themes to justify its inclusion.

2. Methodological Transparency

- Comment: Limited details on how researchers resolved coding discrepancies.

- Recommendation: Clarify the process of resolving disagreements during coding (e.g., consensus meetings, third-party arbitration) to enhance dependability.

3. Ethical and Reproducibility Considerations

- Comment: Data availability is restricted due to GDPR, which is understandable but limits reproducibility.

- Recommendation: Provide a more detailed anonymized raw data summary (e.g., de-identified quotes or themes) in supplementary materials to improve transparency.

5. Conflict of Interest (COI)

- Comment: While COIs are declared, the potential influence of pharmaceutical funding (e.g., Bayer, Pfizer) on interpretation is not addressed.

- Recommendation: Add a brief statement affirming that funders had no role in study design, data interpretation, or manuscript preparation.

**Minor Revisions**

1. Abstract: Clarify the “unrestricted grant” source in the funding statement.

2. Results:

- Define abbreviations at first mention (e.g., “OAC” in Table 1).

- Standardize participant identifiers (e.g., “Ma (38)” vs. “K (79)”—ensure consistency in pseudonyms).

3. Discussion:

- Expand on how cultural context (Danish healthcare system) may influence follow-up care perceptions.

- Compare findings to similar qualitative studies (e.g., Danielsbacka et al., 2021) to contextualize originality.

**Recommendation**: Accept with Minor Revisions
